# CD123 as a Biomarker in Hematolymphoid Malignancies: Principles of Detection and Targeted Therapies

**DOI:** 10.3390/cancers12113087

**Published:** 2020-10-23

**Authors:** Hanadi El Achi, Edouard Dupont, Shilpa Paul, Joseph D. Khoury

**Affiliations:** 1 Department of Pathology and Laboratory Medicine, The University of Texas at Houston, Houston, TX 77030, USA; hanadi.s.elachi@uth.tmc.edu; 2 Faculty of Pharmacy of Paris, Paris Descartes University, 75270 Paris, France; edouard.dupont@etu.parisdescartes.fr; 3 Department of Leukemia, The University of Texas MD Anderson Cancer Center, Houston, TX 77030, USA; SPaul1@mdanderson.org; 4 Department of Hematopathology, The University of Texas MD Anderson Cancer Center, Houston, TX 77030, USA

**Keywords:** CD123, targeted therapy, flow cytometry, immunohistochemistry, biomarker

## Abstract

**Simple Summary:**

CD123 is overexpressed in multiple hematologic malignancies. Advances in CD123-targeted therapies over the past decade have positioned this molecule as an integral biomarker in current practice. This review provides an overview of CD123 biology and in-depth discussion of clinical laboratory techniques used to determine CD123 expression in various hematolymphoid neoplasms. In addition, we describe various pharmacologic strategies and agents that are available or under evaluation for targeting CD123.

**Abstract:**

CD123, the α chain of the interleukin 3 receptor, is a cytokine receptor that is overexpressed in multiple hematolymphoid neoplasms, including acute myeloid leukemia, blastic plasmacytoid dendritic cell neoplasm, acute lymphoblastic leukemia, hairy cell leukemia, and systemic mastocytosis. Importantly, CD123 expression is upregulated in leukemic stem cells relative to non-neoplastic hematopoietic stem cells, which makes it a useful diagnostic and therapeutic biomarker in hematologic malignancies. Varying levels of evidence have shown that CD123-targeted therapy represents a promising therapeutic approach in several cancers. Tagraxofusp, an anti-CD123 antibody conjugated to a diphtheria toxin, has been approved for use in patients with blastic plasmacytoid dendritic cell neoplasm. Multiple clinical trials are investigating the use of various CD123-targeting agents, including chimeric antigen receptor-modified T cells (expressing CD123, monoclonal antibodies, combined CD3-CD123 dual-affinity retargeting antibody therapy, recombinant fusion proteins, and CD123-engager T cells. In this review, we provide an overview of laboratory techniques used to evaluate and monitor CD123 expression, describe the strengths and limitations of detecting this biomarker in guiding therapy decisions, and provide an overview of the pharmacologic principles and strategies used in CD123-targeted therapies.

## 1. Introduction

CD123 has emerged in recent years as an attractive novel target of therapy. CD123 is normally expressed on hematopoietic stem cells (HSC) [1]. Its upregulation in leukemia brought it to the forefront as a target that can be selectively inhibited in neoplastic cells [2,3,4]. Indeed, CD123 expression has been demonstrated on neoplastic cells in blastic plasmacytoid dendritic cell neoplasm (BPDCN), acute myeloid leukemia (AML), acute lymphoblastic leukemia/lymphoma (ALL), hairy cell leukemia (HCL), systemic mastocytosis (SM), etc. [1,5].

Efforts to target CD123 have resulted in the development of agents that effectively target neoplastic cells expressing this molecule. Several drugs designs have been explored in furtherance of this goal, mostly utilizing a humanized anti-CD123 antibody moiety fused with a toxic payload. In parallel, cellular therapy approaches using CD123-targeting chimeric antigen receptor (CAR) T cells have emerged as another exciting approach [6,7]. As these therapies require knowledge of the expression status of CD123 at baseline and during the course of treatment, clinical laboratory techniques for measuring CD123 expression as a biomarker of therapy have gained unprecedented urgency. Arguably, without such sensitive, specific, and reproducible laboratory techniques, treatment decisions involving CD123-targeted therapies would be compromised.

In this review, we provide an overview of CD123 biology, discuss current clinical laboratory techniques used to evaluate CD123 expression on neoplastic cells and salient features of CD123 expression in various hematolymphoid neoplasms, and discuss the major classes of targeted CD123-targeting agents available at present.

## 2. Biology of CD123

CD123 is the alpha chain of the interleukin 3 receptor (IL-3Rα). IL-3R is the IL-3-specific member of the beta common (β_c_) family of receptors, which also includes IL-5R and granulocyte-monocyte colony stimulating factor (GM-CSF) receptor. This family of membrane receptors regulates the growth, proliferation, survival, and differentiation of hematopoietic cells, along with immunity and inflammatory response [8,9,10,11]. Interleukin 3 (IL-3) is a soluble and pleiotropic cytokine that regulates the function and production of hematopoietic and immune cells [12,13]. IL-3 binds to the ligand-specific alpha subunit, resulting in heterodimerization with the common/shared signal-transducing beta (β_c_) subunit.

The CD123 protein is composed of three extracellular domains (287 amino acids), a single-pass transmembrane domain (30 amino acids), and a short intracellular region (53 amino acids) [14,15]. The extracellular domains consist of fibronectin type III (FnIII) components, an Ig-like N-terminal domain (NTD), domain 2 (D2), and domain 3 (D3) [13,14]. D2 and D3 constitute the cytokine receptor module, which is responsible for cytokine binding, while the NTD is responsible for ligand recognition, receptor dimerization, and signaling. Indeed, the NTD appears to have dual roles, the regulation of IL-3 binding and the prevention of spontaneous receptor dimerization [13,14,16]. The gene encoding CD123−*IL3RA*−has been mapped to the X-Y pseudo-autosomal regions of the human sex chromosomes at bands Xp22.3 and Yp11.3, which are notably located near the *CSF2RA* gene encoding the alpha chain of the GM-CSF receptor [17]. Interestingly, the overlap in the genomic structures of the *IL3RA* and *CSF2RA* genes suggest common ancestry in the evolution of the cytokine receptor superfamily, with features indicating that they might have evolved separately from the *ILR5A* gene [18].

**Figure 1:**
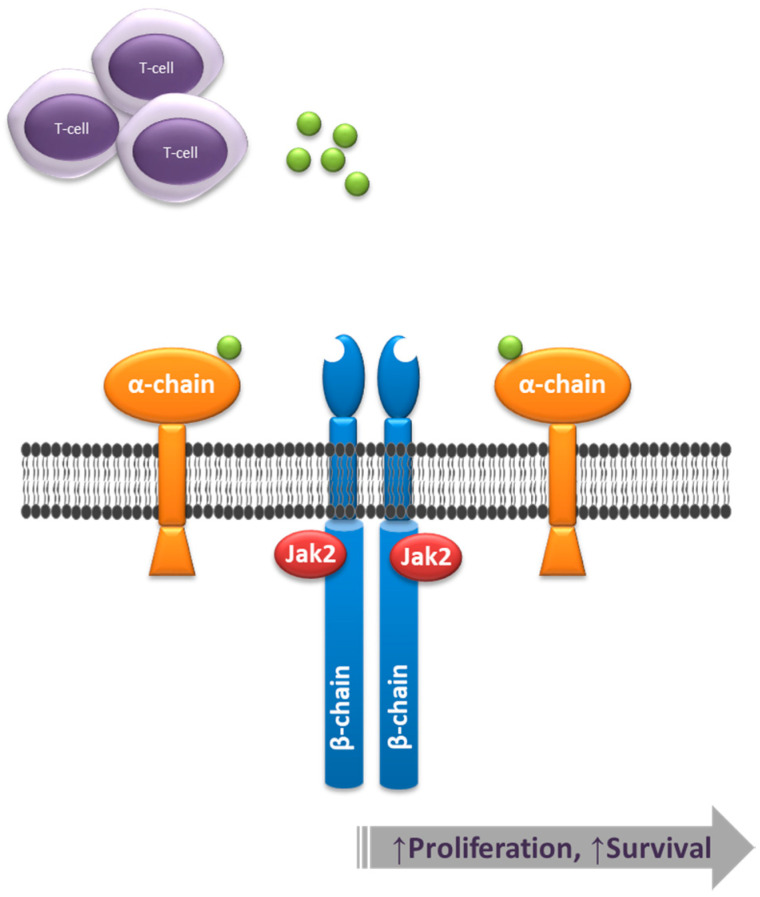
CD123 is the alpha chain of the interleukin 3 receptor (IL-3R). Upon ligand (green moieties) binding, the IL-3R heterodimer comprised of alpha and beta chains signals through Jak2, leading to a downstream activation of effectors that result in a net increase of cell proliferation and survival.

IL-3 binding to CD123 recruits the β_c_ subunit (CD131) to form a high-affinity receptor. Then, the receptor cascades intracellular downstream signals that in turn activate the JAK/STAT, Ras-MAPK, and phosphatidylinositol 3-kinase pathways [19] (Figure 1). It should be noted that in addition to promoting cell proliferation and survival, IL-3 binding, along with GM-CSF, modulates the levels of chemokine stromal cell-derived factor-1 (SDF-1) and its receptor CXCR4, which play a role in the homing and egress of bone marrow hematopoietic cells [20,21]. Indeed, CD123 overexpression in AML results in increased proliferation and enhanced survival, as well as bone marrow egress resulting from downregulation of the SDF-1/CXCR4 axis [21].

## 3. Principles of Laboratory Evaluation of CD123

Assessment of CD123 expression in clinical practice is performed using flow cytometry (FC) or immunohistochemistry. Similar to any laboratory assay, each of these techniques entails inherent strengths and limitations. 

### 3.1. Flow Cytometry

**Figure 2:**
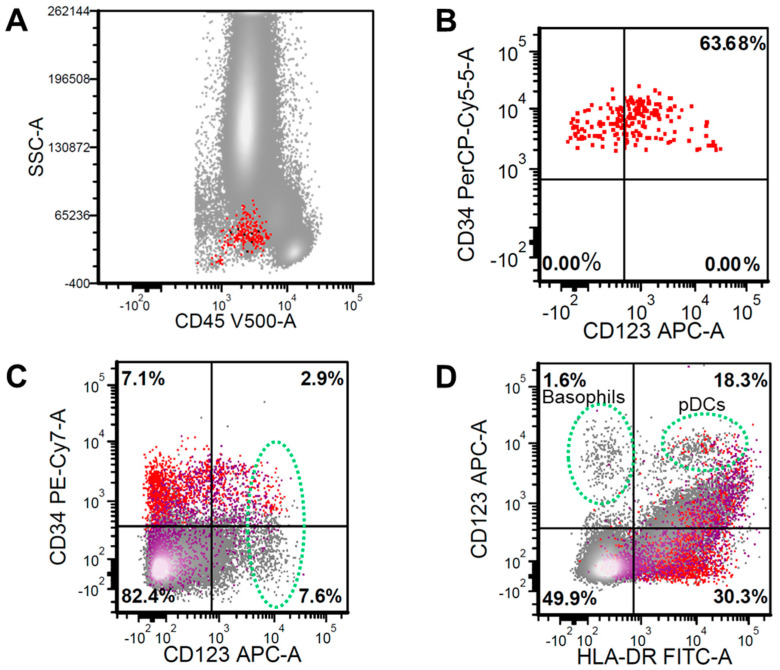
(**A**) Flow cytometry analysis on a bone marrow aspiration sample demonstrating CD34+/CD45^dim^ hematopoietic stem cells (HSC) (red color) (**B**) with normal CD123 expression pattern. (**C**) Combinatorial gating depicts various CD123^bright^ cells (green oval), which include CD34+ HSC elements and CD34− populations (gray), with only rare CD117+ cells (purple). (**D**) CD123^bright^ cells can be further subtyped into basophils (CD123^bright^/HLA−DR−) and plasmacytoid dendritic cells (pDCs) (CD123^bright^/HLA−DR+).

Flow cytometry immunophenotyping is a robust and versatile immunophenotyping technique for hematolymphoid malignancies. Using live cells, fluorescence-tagged antibodies, laser light excitation sources, and sensitive photon detectors, modern flow cytometers are capable of detecting a variety of cellular characteristics, including cell size, cytoplasmic complexity, and protein expression levels. Among the salient features of multicolor FC immunophenotyping is its dynamic (logarithmic) range in terms of antigen detection and its ability to allocate expression to defined cell populations. CD123 expression assessment by FC is a mainstay of clinical assessment for targeted therapy eligibility. The number of CD123 molecules expressed on various cell populations are indirectly reflected in the intensity of fluorescence detected, which is typically expressed as median fluorescence intensity (MFI). Such intensity is normalized against background fluorescence and/or control cell populations [22]. Furthermore, by employing carefully selected lineage and maturation-associated markers, CD123 expression on neoplastic cells can be clearly distinguished from that on other cell populations within a given sample. While this might not be critical in samples with a heavy disease burden, it is particularly important in the context of minimal/measurable residual disease evaluation [23] (Figure 2).

For all its advantages, FC has some inherent limitations. Most notable is its reliance on a fresh sample with adequate viability. While this might not be a factor in settings where FC lab is located in close proximity to the sample collection source, it might pose some logistic difficulties in settings where sample transportation is needed. Another limitation is the need for high proficiency in FC, both technical and interpretive. As such, sites that have limited case numbers might require higher levels of proficiency maintenance. Other limitations include a loss of tissue architecture in the context of FC evaluation, lack of antibody panel standardization, and restricted billing charges.

### 3.2. Immunohistochemistry

**Figure 3:**
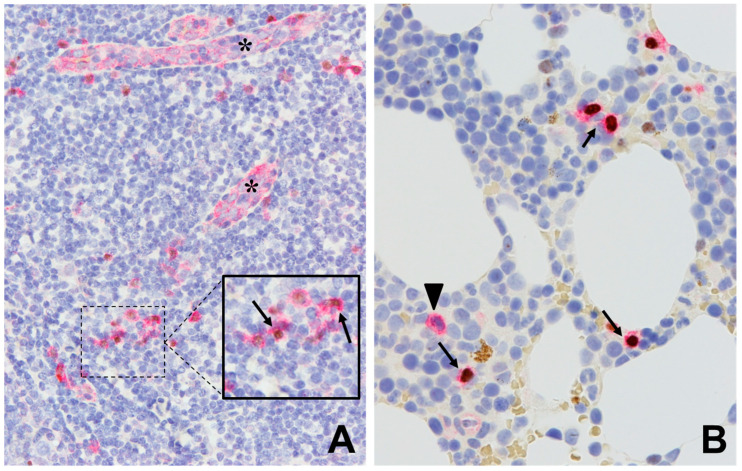
Immunohistochemistry using antibodies against CD123 (red signal, surface/cytoplasmic) and TCF4 (brown signal, nuclear). (**A**) Normal tonsil showing CD123+/TCF4− expression on endothelial cells (asterisks), with a few scattered plasmacytoid dendritic cells showing CD123+/TCF4+ expression (arrow). (**B**) Bone marrow with scattered CD123+/TCF4− myeloid cells (arrowhead) as well as CD123+/TCF4+ plasmacytoid dendritic cells (arrows). (**A** and **B**. Main figures at 200× magnification (inset, 400×), hematoxylin counterstain).

Immunohistochemistry is the mainstay immunophenotyping technique in tissue samples. It permits antigen assessment in virtually any formalin-fixed paraffin-embedded sample type, including percutaneous core biopsy, surgical samples, and decalcified bone marrow samples. This also includes cell block preparations from fine needle aspiration and bone marrow aspirate samples. Modern immunohistochemistry is performed on automated stainers and use complex antigen retrieval and signal development chemistries, which are factors that have improved substantially its sensitivity, specificity, and reproducibility [24,25]. Such innovations have also permitted easier implementation of dual-color immunohistochemistry. CD123 expression assessment by immunohistochemistry is common in tissue biopsy samples involved by neoplasms known to express CD123 [11,26]. The protein is expressed in a restricted manner in tissue components, most notably by endothelial cells and, alongside TCF4, plasmacytoid dendritic cells (pDC). CD123 immunohistochemistry is indispensable in skin biopsy samples suspected of involvement by BPDCN [27]. To improve the specificity of CD123 expression, TCF4 coexpression has been proposed as a coexpression marker in proliferations derived from pDC, including BPDCN [26] (Figure 3).

The limitations of immunohistochemistry are mostly related to its lower sensitivity to low-level CD123 expression in comparison to FC. While the dynamic range of CD123 detection by immunohistochemistry is reasonably broad, it remains unable to detect expression in hematopoietic precursors, for example. 

## 4. CD123 Expression in Hematologic Malignancies

### 4.1. Myeloid Neoplasms

#### 4.1.1. Blastic Plasmacytoid Dendritic Cells Neoplasm

**Figure 4:**
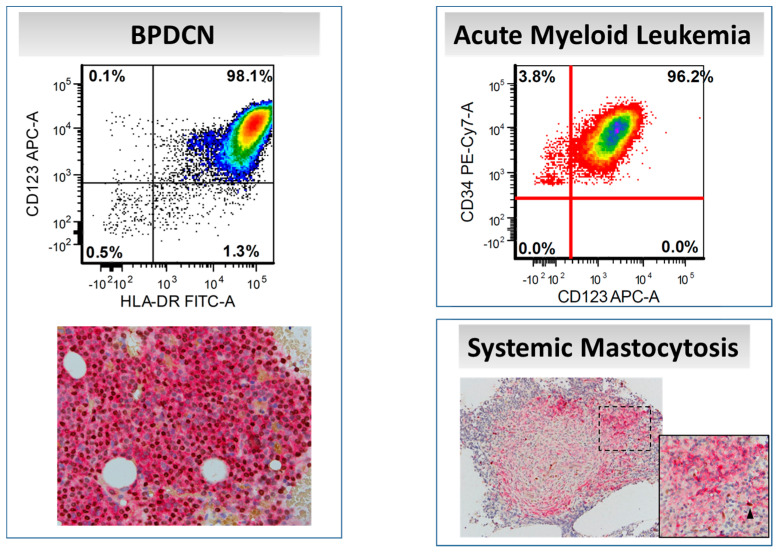
CD123 expression in various myeloid neoplasms, as assessed by flow cytometry and immunohistochemistry (CD123/TCF4 dual-color stain). In systemic mastocytosis, neoplastic mast cells express CD123 (red signal) without TCF4 (brown signal); a few admixed plasmacytoid dendritic cells with CD123/TCF4 coexpression can be seen (arrowhead). (Main figures at 200× magnification (inset 400×), hematoxylin counterstain).

Blastic plasmacytoid dendritic cells neoplasm is a highly aggressive hematologic malignancy derived from pDCs. The disease has been reported in all age groups, but most patients are in the sixth decade of life. Over 80% of patients present with skin lesions with or without systemic involvement that most often includes the bone marrow and lymph nodes [27]. The diagnosis of BPDCN hinges on the detection of CD123 and TCF4, with an absence of lineage-specific markers such as CD3, CD19, CD64, and myeloperoxidase [28] (Figure 4). Most BPDCN cases are positive for CD4, CD56, and TCL1 [29]. Proliferations of mature pDCs have also been described, particularly in association with chronic myelomonocytic leukemia (CMML) [30].

#### 4.1.2. Acute Myeloid Leukemia

Different studies showed CD123 to be expressed in 45–95% of AML cases [2,12,31]. CD123 overexpression in AML has been reported to correlate with mutations in *NPM1* and *FLT3* [32,33,34]. However, notably, the prognostic impact of CD123 appears to be independent of the presence of concurrent genetic alterations known to be involved in therapy resistance [35]. CD123 overexpression on AML blasts has been associated with a negative prognosis with decreased overall survival (OS) and lack of clinical remission (CR) [5,31]. The impact of CD123 overexpression on clinical outcomes in AML is postulated to be related to increased sensitivity to IL-3 binding and to downregulation of CXCR4 (see above) [21]. CD123 overexpression also leads to constitutive STAT5 activation resulting in higher cycling activity and worsening resistance to apoptotic stimuli [31]. 

Cytokines and the cytokine receptors system have been shown to play an important role in leukemogenesis as well as in the biological and clinical behavior of various myeloid neoplasms. In AML, the levels of CD123, GM-CSF, interleukin-2 receptor alpha chain (IL-2Rα; CD25), common gamma-chain cytokine (γc), c-kit, and granulocyte colony-stimulating factor receptor (G-CSFR) were widely expressed with a range of more than 10,000 sites/cell, and their expression patterns correlated with morphologic subtypes [36,37]. 

#### 4.1.3. Myelodysplastic Syndrome

Yue et al. [38] measured the expression of CD123 on CD34+/CD38− cells in the bone marrow of Myelodysplastic Syndrome (MDS) patients and detected overexpression in 48% of cases, which was correlated with the percentage of bone marrow CD34+ blasts. Li and colleagues further showed that the extent and intensity of CD123 expression in high-grade MDS was similar to that in AML [39]. In an attempt to elucidate the role of CD123 in the pathogenesis of MDS and disease progression, Stevens et al. [40] showed that CD123+ on CD34+ hematopoietic stem cells in MDS exhibited significant changes in cellular energy metabolism and higher levels of protein synthesis [40]. CD123 has been proposed as a potential therapeutic target in MDS [41].

#### 4.1.4. Systemic Mastocytosis

CD123 is not expressed normally by mast cells. However, the majority of neoplastic mast cells exhibit aberrant CD123 expression. Pardanani and colleagues studied 58 SM cases, including indolent SM, aggressive SM, SM with associated hematological neoplasm, and mast cell leukemia; CD123 was detected by immunohistochemistry in 100%, 61%, 57%, and 0% of cases, respectively [42]. Other groups identified CD123 and HLA-DR expression in aggressive variants of SM, including mast cell leukemia [43]. Moonim and colleagues reported no association between CD30/CD123 expression and disease aggressiveness in SM [44]. Another interesting finding is the presence of pDCs in association with neoplastic mast cells in CD123+ SM cases [42] (Figure 4). It has been suggested that pDCs may play a role in the pathogenesis of SM [45]. Hence, CD123-targeted therapy would be an interesting option in SM that will attack the tumor cells as well as its microenvironment.

#### 4.1.5. Chronic Myeloid Leukemia

The use of various generations of tyrosine kinase inhibitor therapy has improved the survival of patients with chronic myeloid leukemia (CML) [46]. However, the accelerated and blast phases of the disease remain high-risk categories, possibly to the limited activity of tyrosine kinase inhibitors against CD34+/CD38− leukemic stem cells. Therefore, multiple studies are being conducted to identify new therapeutic agents targeting CML stem cells [47]. The expression of CD123 was found on CD34+/CD38− cells in CML patients, and the level of expression increased with disease progression [48]. These findings are raising the specter of using Tyrosine Kinase Inhibitors (TKI) in combination with anti-CD123 therapy in patients with high-risk CML. The role of IL-2 and IL-2R (CD25) has also been highlighted in CML. High-level CD25 expression has been identified on CD34+/CD38− cells, suggesting that targeting CD25 might provide another avenue to complement TKI treatment to improve CML outcomes [49].

### 4.2. Lymphoid Neoplasms

#### 4.2.1. Acute Lymphoblastic Leukemia/Lymphoma

B acute lymphoblastic leukemia/lymphoma (B-ALL) is typically categorized based on the presence of t(9;21)(q34;q11)/*BCR-ABL1* resulting in the Philadelphia (Ph) chromosome derivative. Cases harboring this translocation are designated as Ph-positive, while those lacking this translocation are designated as Ph-negative. In recent years, a subset of Ph-negative B-ALL has been shown to express a “Ph-like” signature imparting a distinct prognostic and therapeutic impact. CD123 is frequently overexpressed in B-ALL. Angelova and colleagues [22] conducted a large study in which CD123 expression was evaluated by FC in various subsets of B-ALL and T-ALL. They demonstrated CD123 overexpression in 96.6% of Ph-positive and 86.3% of Ph-negative B-ALL. Within the Ph-negative subset, CD123 appears to be more commonly expressed in cases expressing a Ph-like signature [50]. Liu et al. reported that CD123 in B-ALL correlated with worse OS [32]. This was supported by another study demonstrating a high risk of relapse in adult B-ALL patients expressing CD123 [51]. In contrast, Al-Mudallal et al. showed that CD123 expression was associated with a good response to induction therapy in pediatric B-ALL cases [52]. On balance, the prognostic impact of CD123 in B-ALL is debatable, and reported differences in outcomes might be related to the treatment protocols and other prognostic factors.

Angelova and colleagues detected CD123 expression in 43.3% of T-ALL, with the vast majority of cases (92.3%) having early T precursor (ETP) or early non-ETP immunophenotype [23(Khogeer, 2019 #136)(Jain, 2016 #134)] [53,54]. Notably, their findings, in line with those reported by others [2], indicated that CD123 expression on leukemic cells in T-ALL was at a lower MFI compared to B-ALL.

#### 4.2.2. Hairy Cell Leukemia

The expression of CD123 on mature B lymphoproliferative disorders in general and B-cell disorders with hairy or villous lymphocytes in particular was extensively investigated. CD123 overexpression has been demonstrated in almost all cases of HCL, with rare expression in HCL variant and splenic lymphoma with villous lymphocytes [55]. Recent reports have further confirmed CD123 overexpression in HCL but showed partial/dim CD123 expression in 40% of HCL variant, 33% of MCL, 33% of FL, 25% of SMZL, and 4% of CLL [56].

#### 4.2.3. Hodgkin Lymphoma

CD123 is expressed on Hodgkin–Reed–Sternberg cells in 90% of Hodgkin lymphoma (HL), and IL-3 is capable of inducing Hodgkin cell line growth in vitro [57]. CD123 overexpression appears to be highest in nodular sclerosis HL [58]. Other data suggest that CD123 expression is also present on macrophages in the microenvironment of HL, suggesting that targeting CD123 might impact both the neoplastic cells as well as the tumor microenvironment [59].

## 5. CD123-Targeted Therapies

**Figure 5:**
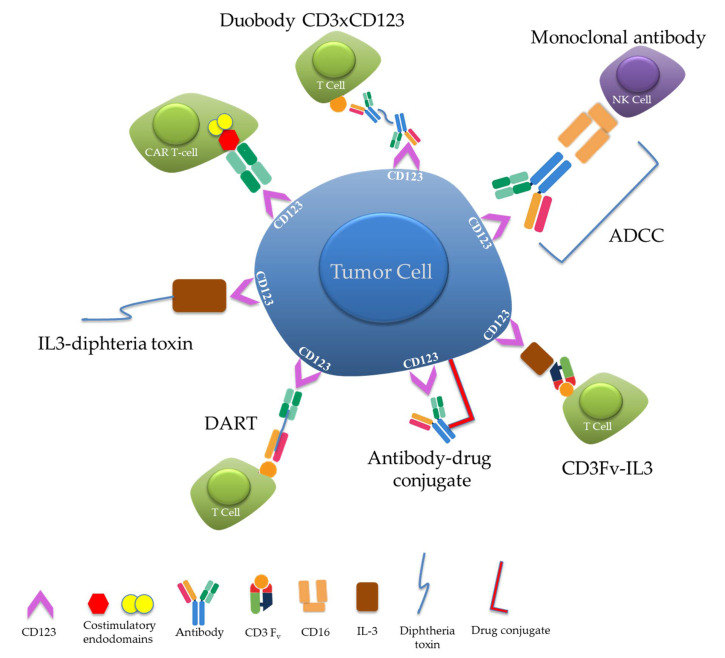
Schematic diagram of various CD123-targeted therapeutic constructs.

A number of CD123-targeted therapies have been developed over the past decades. The ensuing section is aimed at providing an overview of design strategies and salient advances in the use of CD123-targeted therapies in patients with various hematolymphoid malignancies. Table 1 provides a summary of the agents discussed herein, while Figure 5 provides a schematic overview of the drug designs employed.

### 5.1. Recombinant Fusion Protein (IL3-Diphtheria Toxin)

In 2000, Frankel and colleagues [60] reported the development of a novel recombinant protein that consisted of the IL-3 fused using a (G4S)2 linker with the catalytic and translocation domains of diphtheria toxin. The product showed a cytotoxic effect on myeloid leukemia cell lines in vitro, and the degree of cytotoxicity was linked to level of CD123 expression [60]. Subsequent optimization confirmed effectiveness against myeloid cells under various in vitro, ex vivo, and clinical settings [61,62]. Currently, numerous clinical trials are exploring the efficiency of tagraxofusp (formerly SL-401) in multiple settings, including relapsed/refractory AML and MDS.

An ongoing clinical trial (NCT02268253) is evaluating the effect of tagraxofusp in patients with myelofibrosis [63]. Preliminary results of the study involving relapsed/refractory patients or those intolerant to Janus Kinase (JAK) inhibitors show a remarkable reduction in splenomegaly in advanced disease [64]. CD123 is expressed in CMML blasts, monocytes, and neoplastic pDCs [65]. Tagraxofusp achieved satisfactory response in treating CMML patients in terms of reduction in spleen size [66].

Frolova et al. [48] confirmed that IL3R is significantly expressed on CD34 (+) BCR-ABL1(+) stem cells in CML cases. Therefore, they explored SL-401 (DT388-IL3) and SL-501 (DT388-IL3 [K116W]) as potential therapeutic options in TKI-resistant CML cases [48]. They reported that combining TKI with recombinant fusion proteins enhanced leukemic cell death [48]. 

The interaction between plasma cell myeloma cells and pDCs stimulate IL-3 expression, which consequently enhances myeloma cells’ development and increases pDC survival [67,68]. Targeting pDCs in multiple myeloma using tagraxofusp blocked the growth of neoplastic cells, decreased serum paraprotein levels, and enhanced the activity of bortezomib and pomalidomide [69,70]. Interestingly, IL-3 receptors on osteoclastic progenitors seem to result in improved halting of bone resorption as a result of tagraxofusp therapy [45,69].

A phase I/II study (NCT00397579) evaluated the response of 11 patients with BPDCN who received tagraxofusp. Five patients had CR and two had partial response after a single course; the duration of clinical response fluctuated between 1 and 20 months (median, 5 months) [71]. Similar outcomes were described in vivo and in vitro [72]. These findings led to a multicenter phase III trial, which included 47 patients with untreated and relapsed BPDCN who received intravenous infusion of tagraxofusp. The response rate among relapsed patients was 67%, and the median OS was 8.5 months. A total of 72% of the untreated patients had a CR. Among those who underwent SCT, the 2-year survival was 52% [73]. Based on these findings, the United States Food and Drug Administration (FDA) approved tagraxofusp-erzs for BPDCN treatment in adults and pediatric patients 2 years and older [74]. The most common adverse effects consist of increased alanine aminotransferase (64%) and aspartate aminotransferase levels (60%), hypoalbuminemia (55%), peripheral edema (51%), and thrombocytopenia (49%). Most importantly, capillary leak syndrome (CLS) was observed in 19%; most of the cases showed moderate severity; however, deaths due to CLS occurred in two patients. Therefore, stringent monitoring of serum albumin, weight, and blood pressure are of utmost importance for patients treated with tagraxofusp [73,75].

### 5.2. Therapeutic Monoclonal Antibodies against CD123

Jin and colleagues [76] reported on an anti-IL-3R neutralizing antibody (7G3) to target AML cells. Clone 7G3 first inhibited the engraftment of leukemic cells in vivo by inhibiting the binding of IL-3 and triggered the activation of the immune system in mice [76]. The fully humanized clone CSL362 showed affinity to Fc gamma receptor IIIA (CD16) on natural killers cells (NK) engendering an enhanced cytotoxic effect against CD123+ cell lines in vitro and naïve AML cells in vivo [77]. The non-Fc-engineered CD123 monoclonal antibody did not induce anti-leukemic activity in AML patients; therefore, monoclonal antibody blockade of CD123 function solely seemed insufficient as a therapeutic strategy [63]. The drug has been tested against AML blasts in combination with cytarabine and daunorubicin and showed a synergistic effect without affective normal HSC [78]. In a phase I trial, CSL362 resulted in 50% of patients being maintained CR after 6 months, and the median duration of CR was 34 weeks [79].

After chemotherapy and transplantation, the number of NK cells is increased in AML patients [80], further enhancing the impact of CSL362 in stimulating NK cell-mediated antibody-dependent cell cytotoxicity (ADCC). The SAMBA trial used talacotuzumab (JNJ-56022473), which is an IgG1 monoclonal antibody against CD123 for the treatment of high-risk MDS or AML patients failing first-line treatment. They reported that patients had significantly less mature NK cells and less activating NK-cell receptors compared to controls. Overall, their results showed moderate benefits of treating relapsed AML and MDS with monoclonal antibodies against CD123 only [81,82].

Although HL cells express CD123, the limited number of NK cells in the microenvironment limits the ADCC induced by talacotuzumab. Ernst and colleagues demonstrated that the combination of CSL362 and high-affinity Fc gamma-receptor NK-92 cells (haNK) effectively target and kill HL cells in vitro [83]. Based on these findings, CD123 monoclonal antibody can be used to treat all hematologic diseases in which CD123 is highly expressed independently of the size of the NK cells clusters. On the other hand, KHK2823, a non-fucosylated fully humanized monoclonal antibody against CD123 was found to bind to cells from AML, MDS, and B-ALL patients [81], and it is currently being investigated in patients with AML and MDS (NCT02181699). Moreover, CD123 expression was high in CD34+/CD38− cells blast and chronic phases of CML, and the level of expression increased with disease progression, as mentioned above. Adding CSL362 to the CML regimen enhanced the effect of the tyrosine kinase inhibitors in eliminating the CML precursors [84].

### 5.3. Bispecific Antibody-Based Molecules

Kuo and colleagues [102] developed a bispecific CD3 × CD123 dual-affinity retargeting therapy (DART) molecule. This drug stimulates the immune system through the activation and expansion of cytokine-secreting T cells [102]. The DART molecule creates a bridge between CD123 on target cells and CD3 on T cells and mediates anti-cancer activity via an enhancement of the cytolytic activity of T cells. Compared with other bispecific antibodies, the DART platform is characterized by high stability due to interchain disulfide bridging at the level of the Fv domain [102]. Moreover, in comparison with the bispecific T cell engager (BiTe) designs, DART achieves a higher magnitude of T cell activation [103]. The ensuing product, flotetuzumab (MGD006), consisted of a bispecific antibody comprised of the V_H_ domain of one antibody and the V_L_ of the other, recognizing the N-extracellular domain of CD123 and the extracellular domain of CD3 within the human T cell receptor complex. [86] The drug resulted in a significant amplification of T cells and the destruction of CD123+ AML cells in vitro and in vivo [104]. Due to the short half-life of flotetuzumab, it is given as a continuous infusion [105].

Godwin et al. [87] studied six patients with refractory high-risk cytogenetic AML, and 50% of the patients achieved CR. The authors reported that CD3 and CD8 T cells were higher in CR patients versus non-responders [87], which is a finding that highlights the pivotal role of the immune system activation in achieving the desired outcomes [87]. The activation of T-lymphocytes resulted in PD-1 induction, engendering high PD-L1 levels in post-treatment biopsy of non-responders compared to responders. These outcomes urged driving trials combining CD123 × CD3 bispecific antibody therapy with anti-PD-1 antibody in the treatment of AML patients [106]. A phase I trial is evaluating the efficacy of flotetuzumab (MGD006/S80880) in relapsed/refractory AML or intermediate to high risk MDS (NCT02152956).

APVO436 consists of two full-length IgG bispecific immunoglobulins that retain the Fc region of the antibody and therefore has higher stability in vivo and a longer half-life compared to DART, thus requiring intermittent instead of continuous infusion (NCT03647800) [105].

JNJ-63709178 is being assessed for the treatment of RR AML (NCT02715011), and XmAb14045 is being studied for the treatment of AML, B-ALL, BPDCN, and CML blast crisis (NCT02730312). Other bi-specific antibodies have been designed to include a novel conjugate of single-chain Fv antibody fragments specific for CD123 with an anti-CD16 antibody to enhance the recruitment of CD16-positive NK cells, thereby inducing better ADCC activity [88].

Recently, triplebody dual-targeting molecules have been developed using triple single-chain Fv antibody fragments (scFvs). These molecules have longer half-lives and can target both CD33/CD123 positive tumor cells as well as CD16 on NK cells, inducing a stronger elimination of leukemic cells in vitro [107]. Braciak and colleagues developed SPM-2, which is a triplebody molecule with binding sites for CD33, CD123, and CD16. Large clusters of CD33 and CD123 on the surface of leukemic cells were needed to achieve the maximum efficiency of this drug [108].

### 5.4. CD3Fv-IL3 Fusion Constructs

The deletion of eight C-terminal amino acid residues from IL-3 engender higher affinity interactions with its receptor CD123 and better anti-leukemic effect in drug constructs [109]. Fan and colleagues used a C-terminal eight-amino-acids-deleted IL-3 combined with anti-CD3Fv to build a fusion protein named antiCD3Fv-⊿IL3 [90]. To improve the stability of the protein, they generated a disulfide-stabilized variant of the product. High-binding capacity resulted in a very specific attack of the CD123-positive cells [90].

### 5.5. CD123 Engager T Cells

This product consists of two single chain variable fragments (scFVs) specific for tumor antigen and for CD3. This construct provides a unique characteristic to destroy tumor cells and attract resident T cells to neoplastic cells. The infusion of genetically modified T cells will allow an accumulation of their recombinant bispecific antibody product in the tumor area, hence prolonging the treatment effect. The engager had significant in vivo cytotoxic activity against tumor cells and notable prolongation in survival [91]. CD123-ENG T cells directed against AML blasts showed similar outcomes. However, the new product is able to lyse normal HSC when the effector-to-target (E:T) ratio is high. Thus, to eliminate this risk, a CD20 suicide gene (CD20.CD123-ENG T cells) was added to the modified infused T cells. The CD20.CD123-ENG T lymphocytes showed the same effect on tumor cells and also allowed rituximab-mediated ENG-T cell elimination when the E:T is high [92]. To improve the engager therapy, Doherty and colleagues engineered a new CD123-ENGs that provides a co-stimulation of T cells to increase their expansion and persistence for better tumor killing. The new product was constructed by genetically modifying the T cells to encode CD123-ENG linked to an inducible costimulatory molecule MYD88/CD40 (CD123-ENG.2A.iMC), which can be triggered by a chemical inducer of dimerization. Testing the molecule in vitro on AML cells showed improved function compared to CD123-ENG by itself [93]. Recently, IL-15 has been shown to enhance the cytotoxic activity of CD8+ T cells and generates memory T cells [94]. Therefore, IL-15 expressing CD123-ENG T cells were developed to improve the role of CD123-ENG T cells. Mu and colleagues generated a CD123-ENG combined with the CD20 suicide gene expressing IL15 (CD20.2A.CD123-ENG.2A.IL15) [95].

### 5.6. CD123-Targeting Antibody-Drug Conjugate

IMGN632 is a CD123-targeting antibody-drug conjugate (ADC) consisting of a humanized anti-CD123 antibody linked to a DNA mono-alkylating cytotoxic payload known as indolinobenzodiazepine pseudodimer. This molecule was tested in vivo and in vitro showing limited toxicity in normal myeloid progenitors [110]. A phase I study in patients with relapsed/refractory AML or BPDCN using IMGN632 showed a manageable safety profile, with side effects including diarrhea (30%), febrile neutropenia (27%), nausea (26%), chills (23%), lung infection (22%), and infusion-related reactions (16%); high doses caused prolonged neutropenia and veno-occlusive disease [111]. SGN-CD123A, another antibody-drug conjugate, uses the pyrrolobenzodiazepine dimer linker and a humanized CD123 antibody. The molecule was evaluated in vitro on AML cell lines and patient samples with FLT3 mutations and/or unfavorable cytogenetic signature; the results showed significant cytotoxic activity and long-lasting CR [96]. Based on these findings, clinical trials to evaluate the efficacy and safety of SGN-CD123 in AML cases are conducted (NCT02848248).

Angelova and colleagues showed that IMGN632 therapy in vitro was effective against B-ALL cell lines and primary B-ALL cells expressing CD123, with no effect on normal lymphocytes [22]. Currently, IMGN632 monotherapy is being investigated in relapsed/refracotry AML, BPDCN, ALL, and other CD123+ myeloproliferative neoplasms (NCT03386513). IMGN632 is also being evaluated in combination therapy with venetoclax and/or azacitidine in CD123+ AML patients (NCT04086264).

### 5.7. Chimeric Antigen Receptor T Cell Therapy (CART)

A CAR is a fusion protein combining an extracellular domain that consists of a single-chain variable fragment (scFv) derived from antibodies, a trans-membrane connecting segment, and an intra-cellular signaling domain. The intracellular domain of first-generation CARs consisted only of the zeta-signal-transducing subunit of the TCR/CD3 receptor complex (CD3z), whereas second- and third-generation CARs possess respectively one or two co-stimulatory endo-domain molecules, such as CD28, OX40, and CD137 fused to CD3z [97]. CAR T cells have multiple characteristics, including a targeted recognition of specific tumor markers, with high avidity and prolonged persistence of transfused T cells providing longer protection. After the success of the ELIANA (NCT02435849) and JULIET (NCT02445248) trials, which investigated tisagenlecleucel, an anti-CD19 CAR T cell construct, for the treatment of pediatric patients with relapsed/refractory B-ALL and adults with relapsed/refractory Diffuse Large B-Cell Lymphoma (DLBCL), respectively, the anti-CD19 CAR T cells have been approved by the FDA [112,113]. Moreover, axicabtagene ciloleucel, which belongs to the same family of immunotherapy constructs, has been approved for the treatment of relapsed large B-cell lymphomas [114]. Some groups have reported the emergence of CD19 downregulation on B-ALL cells after CD19 CART treatment [115].

CD123 is an attractive target for CART in AML; however, since CD123 is dimly expressed on some normal hematopoietic cells and endothelial cells, undesirable off-target effects can result [98]. In addition, responses are not sustained; therefore, allogeneic SCT may be necessary, if feasible. Other researchers reported a potent anti-leukemic effect of the second and third generation CD123 CART with limited inhibition of normal bone marrow progenitors [6,99]. The discrepancy in the results can be related to the source of stem cells used in the experiment; the latter used human adult bone marrow as HSC; however, others used fetal liver, which shows high levels of CD123 expression [98]. Severe cytokine release syndrome and capillary leak syndrome were described in the patients with AML and BPDCN who were treated with an anti-CD123 “universal” CART (UCART123) as part of Phase 1 clinical trials (NCT03203369, NCT03190278 respectively). One fatality was reported with the BPDCN trial of a 78-year-old patient. These findings led to discontinuation of the trial by the FDA [116]. Then, the study was resumed after adjustment of the UCART123 dose [117]. To prevent earlier toxicities from CD123 CART, Cummins and colleagues generated biodegradable CART123 cells with a limited capacity to proliferate (NCT02623582). Although no adverse events have been reported, an anti-tumor effect could not be demonstrated [118]. Furthermore, if an emergent elimination of CAR T cells were needed due to severe cytokine release syndrome, researchers developed a “kill-switch” with the addition of a CD20 marker on the engineered 123-CAR T cells (CAR T 123-CD20), which can be targeted with rituximab [119].

A phase I clinical trial (NCT02159495) is examining the safety and efficacy of CD123 CAR T cell therapy using a lentivirally transduced second-generation CAR with CD28 co-stimulation (CD123CAR-CD28-CD3ζ-EGFRt) in adult patients with relapsed/refractory AML and persistent or recurrent BPDCN. Clinical remission has been achieved in patients treated with low doses of CD123 CART (MB-102) without evidence of dose-limiting side effects [120]. Based on these findings, the FDA has granted an orphan drug designation to MB-102 (CD123 CAR T cell) for AML and BPDCN [100].

Furthermore, a CAR lentiviral vector containing the CD123CAR-CD28-CD3ζ-EGFRt was designed with CD123+ hematopoietic stem cells in MDS. CAR T cells were able to attack CD123+ cells with an antigen density of >1 × 10^4^ with sparing of normal hematopoietic stem cells that express lower antigen densities [121].

The applications of CD123CART have expanded to encompass treatment-resistant HL cases. Ruella et al. demonstrated that the CD123 CART could overcome the immunosuppressive tumor microenvironment of HL and eliminate the HC [59].

## 6. Conclusions

The tremendous advances achieved recently in understanding of cancer pathogenesis allowed the emergence of new biomarkers that can have significant diagnostic, prognostic, and therapeutic implications. CD123 has gained particular attention due to its widespread expression on numerous hematolymphoid malignancies, particularly AML and BPDCN. Promising CD123 targeted therapies have been developed and continue to be examined, ensuring the continued need to evaluate CD123 expression as an integral biomarker whose presence represents a targetable vulnerability.

## Figures and Tables

**Table 1:** Current agents and clinical trials exploring CD123 as a marker for targeted therapy.

Agent	Mechanism of Action	Targetable Diseases	Clinical Trial	FDA Approval Status	References
**Tagraxofusp**	Recombinant fusion protein (IL3-diphtheria toxin)	AML/MDS	NCT02113982 NCT02270463 NCT03113643	n/a	[61,62]
		CML		n/a	[48]
		MF	NCT02268253	n/a	[64]
		CMML	NCT02268253	n/a	[65]
		MM	NCT02661022	n/a	[68,69,70]
		BPDCN	NCT00397579	Approved	[72,73]
**Talacotuzumab**	Anti-IL-3 receptor neutralizing antibody	AML/MDS	NCT02472145 NCT03011034	n/a	[78,79,82,85]
		Hodgkin lymphoma B-ALL CML		n/a	[81,82,83]
**Flotetuzumab**	CD 3 × CD123 DART	AML MDS	NCT02152956	n/a	[86,87]
**APVO436**	Full-length IgG bispecific immunoglobulins (CD123 × CD3)	AML MDS	NCT03647800	n/a	[88]
**JNJ-63709178**	Full-length IgG bispecific immunoglobulins (CD123 × CD3)	AML	NCT02715011	n/a	[82,85]
**XmAb14045**	Full-length IgG bispecific immunoglobulins (CD123 × CD3)	AML B-ALL BPDCN CML Blast Crisis	NCT02730312	n/a	[89]
**Anti-CD3Fv-** **⊿IL3**	CD3Fv-IL3 fusion constructs	AML		n/a	[90]
**CD123-ENG T cells**	In situ secretion of bispecific anti-CD123 × anti-CD3 diabodies by genetically modified T cells	AML		n/a	[91,92,93,94,95]
**IMGN632**	Humanized anti-CD123 antibody linked to a DNA mono-alkylating payload	AML	NCT02848248	n/a	[22,96]
		AML BPDCN ALL MPN	NCT03386513		
**UCART123**	CART cells	AML	NCT03203369	Orphan drug designation for AML and BPDCN	[6,7,97,98,99,100,101]
		HL	NCT02159495		

Abbreviations: FDA: Food and Drug Administration. AML: Acute Myeloid Leukemia. MDS: Myelodysplastic Syndrome. BPDCN: Blastic Plasmacytoid Dendritic Cell Neoplasm. ALL: Acute Lymphoblastic Leukemia. HL: Hodgkin Lymphoma. CML: Chronic Myeloid Leukemia. CMML: Chronic Myelomonocytic Leukemia. MF: Myelofibrosis. DART: Dual Affinity Retargeting. UCART: Universal Chimeric Antigen Receptor T-cell.
